# A person-oriented approach to social anxiety and depression: latent profiles and emotional functioning in adults

**DOI:** 10.3389/fpsyg.2025.1600531

**Published:** 2025-10-20

**Authors:** Jianan Zhou, Nejra van Zalk

**Affiliations:** Dyson School of Design Engineering, Imperial College London, London, United Kingdom

**Keywords:** latent profile analysis, social anxiety, depressive symptoms, daily affect, emotion regulation

## Abstract

**Objectives:**

Symptoms of social anxiety and depression often co-occur, but many questions remain about symptom-level co-occurrence and the heterogeneity of symptom presentations across individuals, as well as their emotional functioning. This study aimed to investigate the co-occurrence of social anxiety and depressive symptoms in adults and variations in emotional functioning linking symptom heterogeneity.

**Methods:**

This study used a person-oriented approach, Latent Profile Analysis (LPA), to identify distinct profiles (i.e., subgroups) in a UK adult sample (*N* = 222) varying in presentations of social anxiety and depressive symptoms. Further analyses examined between-profile differences in emotional functioning, including daily affect and emotion regulation.

**Results:**

Four profiles were identified: Comorbid (12.61%), Dysphoric (10.36%), Socially Anxious (36.94%), and Low Distress (40.09%), replicating the four-profile solution revealed in prior research on adolescents. The Comorbid subgroup reported the most pronounced emotional dysfunction, with higher daily negative affect, lower positive affect, and greater emotion dysregulation than the other three subgroups. The Low Distress subgroup reported the best emotional functioning.

**Conclusions:**

The cross-sectional study design restricts our ability to evaluate the long-term stability of the identified profiles. Nevertheless, this study illuminates the diverse ways social anxiety and depression intertwine, underscoring the necessity of transdiagnostic interventions that cater to a wide range of symptom patterns and emotional functioning.

## 1 Introduction

Social anxiety and depression are highly prevalent emotional disorders that frequently co-occur (Saha et al., 2021) and demonstrate one of the strongest inter-disorder associations (Kessler et al., 2005; Langer and Rodebaugh, 2014). Their comorbidity significantly amplifies the risks for greater symptom severity, higher risks of alcohol abuse and suicide, and more complex treatment requirements compared to individual disorders (Adams et al., 2018, 2016; Dalrymple and Zimmerman, 2007; Koyuncu et al., 2019, 2014; Lecrubier and Weiller, 1997; Norton et al., 2008). Beyond diagnostic-level overlap, these conditions also frequently intersect at the symptomatic level (Epkins and Heckler, 2011; Flynn et al., 2019). In this study, we investigate the symptom-level co-occurrence of social anxiety and depression in an adult community sample. Although defined categorically for clinical purposes, emotional distress manifests on a continuum across the population (Morris et al., 2022). Adopting a dimensional perspective can capture the full spectrum of symptom severity, including subthreshold distress (Cohen et al., 2014). Epidemiological surveys have consistently identified a significant number of individuals in the general population experiencing attenuated psychiatric symptoms (Bosman et al., 2019; Johnson et al., 2022; Koummati et al., 2025). However, individuals with subthreshold distress tend to show functional status and clinical outcomes more similar to those of clinically diagnosed individuals than to those of healthy controls (Kroenke, 2006; Lyness et al., 2006), and subthreshold distress is a significant precursor to and a risk factor for later disorders (Zhang et al., 2023). Therefore, it is crucial to identify mild symptoms to prevent their progression toward the pathological end of the continuum. Additionally, it is essential to distinguish them from the other end without risking pathologizing normal adaptive responses to life events and stressors (Helmchen and Linden, 2000). Accordingly, while research on disorder comorbidity can provide valuable clinical insights, this study focuses on the symptom-level co-occurrence to broaden understanding of population-level patterns and mental health associations for individuals outside the clinical extremes (Kircanski et al., 2017).

Research on comorbidity has traditionally relied on *variable-oriented* analyses to investigate the structure of psychopathology using group averages to study the presentations, associations, and implications of co-occurring disorders (Kim and Eaton, 2017). However, Piccirillo and Rodebaugh (2022) found that patterns of comorbidity between social anxiety and major depression vary substantially across individuals, with significant symptom-level heterogeneity. *Person-oriented* analytic approaches provide a means to account for such heterogeneity (Olino et al., 2012). Latent Profile Analysis (LPA) is a useful person-oriented approach that enables rigorous profile selection compared to typical clustering methods through its model-driven evaluation (Pastor et al., 2007). Instead of assuming the sample represents a uniform population with a single set of averaged characteristics, LPA identifies distinct, homogeneous profiles (i.e., subgroups) within a sample by grouping individuals based on similar responses to measures of interest (also called LPA indicators) (Masyn, 2013; Nylund, 2007). The identified subgroups reflect naturally occurring symptom configurations. Furthermore, as LPA is inherently data-driven, it necessitates adequate variation in symptom severity to facilitate the emergence of distinct profiles, which is particularly well-suited to community samples (Williams and Kibowski, 2015) that encompass a broad continuum of symptom severity (from minimal to clinically significant levels), thereby allowing for the identification of subgroups reflecting real-world distributions. In contrast, a variable-centered approach to understanding the interplay between symptoms of social anxiety and depression, albeit useful, may provide overly simplistic explanations of their co-occurrence by focusing on overall associations rather than subgroup-specific patterns.

LPA has demonstrated effectiveness at detecting heterogeneity in symptom co-occurrence linking symptoms of depression with generalized anxiety (Dai et al., 2024; Morales-Muñoz et al., 2023; Wang et al., 2021) and PTSD (Contractor et al., 2017; Kline et al., 2024; Zhen et al., 2019), but studies focusing on symptoms of social anxiety and depression are scarce. To our knowledge, only one study, Magson et al. (2022), used LPA to explore the co-occurrence of social anxiety and depressive symptoms in a Swedish adolescent sample, identifying four subgroups: *comorbid* (high levels of social anxiety and depression), *dysphoric* (moderate depression, low social anxiety), *socially anxious* (moderate social anxiety, low depression), and *low distress* (low levels of both symptoms). Several other studies have identified latent profiles based on symptoms of social anxiety and depression, but in conjunction with other characteristics. For example, Liang et al. (2023) identified five profiles of social anxiety and depression in combination with loneliness among elementary school students, including congruent-low, moderately low with predominant loneliness and depressive symptoms, moderate with predominant loneliness and depressive symptoms, moderately low with predominant social anxiety, and congruent-high. Käll et al. (2021) identified five profiles based on loneliness and a combination of social anxiety, depression, and worry: low levels of all indicators, moderate depression and worry, primarily socially anxious, moderate levels, and high levels of all indicators. Focusing on loneliness as well as social anxiety, depression, and paranoia among young adults, Chau et al. (2022) identified five profiles ranging from low levels of all indicators to elevated loneliness accompanied by heightened levels of each symptom. Moreover, Fitzke et al. (2024) identified four profiles among heavy-drinking college students, including low social anxiety and depressive symptoms with low pregaming motives, moderate symptoms and motives, subclinical symptoms with high motives, and clinically elevated symptoms with moderate motives. Although valuable, these studies incorporate additional indicators in their profiling, making it challenging to isolate the specific co-occurrence of social anxiety and depressive symptoms. In addition, the person-oriented studies to date have primarily focused on younger populations, including children, adolescents, and young adults. Further research is necessary to ascertain whether the symptom profiles and prevalence observed in younger individuals also manifest in adults, as well as investigating the underlying factors that influence symptoms of social anxiety and depression throughout the lifespan.

There are additional compelling reasons to use a person-oriented approach in studying the comorbidity of social anxiety and depressive symptoms. Individuals with social anxiety and depression, respectively, share emotional functioning difficulties, including higher negative affect and lower positive affect (Chin et al., 2023; Rum et al., 2024; Wang et al., 2012) as well as emotion dysregulation, such as greater rumination (Arditte Hall et al., 2019; Grant et al., 2014) and maladaptive use of cognitive reappraisal and emotional suppression (Dryman and Heimberg, 2018). However, most research on emotional functioning has used variable-oriented approaches, focusing on mean-level differences rather than analyzing individuals as distinct units and exploring differences within specific subgroups.

Previous work has employed LPA to examine the associations between psychiatric symptoms and emotional functioning, though not focusing on social anxiety and depression *per se*. Lei et al. (2022) showed the associations of distinct patterns of state anxiety and depressive symptoms with negative and positive affect. The profile with high levels of both symptoms reported the highest negative affect, while the minimal symptom profile had the lowest. The depressive/moderately anxious and mildly anxious profiles exhibited intermediate levels. Positive affect differences were more subtle, with only the minimal symptom profile significantly higher than the depressive/moderately anxious profile. In addition, Kökönyei et al. (2024) revealed significant differences in emotion regulation between the depression profiles, with the moderate and high symptom subgroups relying more on maladaptive emotion regulation strategies and using fewer adaptive strategies compared to the low depression subgroup. Alternatively, some studies have explored the heterogeneity in emotion regulation and associated these regulation profiles with psychiatric symptoms. For example, Liu et al. (2024) found that the subgroup experiencing significant emotion regulation difficulties exhibited higher levels of generalized anxiety, depression, and psychological distress compared to the other two subgroups with fewer or minimal regulation difficulties. McGlinchey et al. (2021), surprisingly, found no difference in the network connectivity of generalized anxiety and depressive symptoms between the low and intermediate emotion regulation profiles. To our knowledge, no studies have specifically explored the variations in emotional functioning linking social anxiety-depressive symptom heterogeneity.

Taken together, significant gaps remain in understanding the symptom-level co-occurrence of social anxiety and depression in adult populations. While LPA has been applied to related psychiatric constructs, only Magson et al. (2022) specifically profiled the co-occurrence of social anxiety and depressive symptoms in adolescents. It is unknown whether the symptom profiles observed in youth generalize to adult populations given developmental differences in social-emotional processing (Steinberg, 2005). Moreover, whether empirically derived social anxiety-depression profiles link to core emotional functioning processes remains unknown. To address these gaps in the existing literature, the current study introduces two key innovations. First, we employ LPA to identify the latent profiles based exclusively on social anxiety and depressive symptoms within a UK adult community sample, providing the first person-oriented examination of social anxiety-depressive symptom heterogeneity in adults. Second, we move beyond profile identification to investigate how distinct symptom profiles associate with emotional functioning (i.e., daily affect and emotion regulation). This investigation not only validates the theoretical and functional significance of data-driven profiles but also reveals profile-specific pathways linking symptom configurations to transdiagnostic emotional processes.

Based on Magson et al. (2022)'s LPA findings in a youth population, we expect to identify four latent profiles in our adult sample with distinct symptom configurations: (1) low levels of social anxiety and depressive symptoms, (2) high levels of both, (3) moderate social anxiety and low depressive symptoms, and (4) moderate depressive symptoms and low social anxiety. Furthermore, by leveraging separate bodies of literature on emotional functioning in social anxiety and depression, we expect to uncover significant profile differences in daily affect and emotion regulation. Compared to profiles characterized by lower severity, those characterized by higher symptom severity are expected to be associated with higher daily negative affect and greater emotion dysregulation (i.e., rumination and suppression), as well as lower positive affect and less adaptive emotion regulation (i.e., reappraisal).

## 2 Materials and methods

### 2.1 Procedure and participants

The study is a secondary analysis of data originally gathered using Qualtrics in November and December 2014 through a survey designed to align with “The Big Personality Test”, a large-scale open online study conducted by the British Broadcasting Corporation (BBC) on its “Lab UK” website. Participation in the original study was voluntary, with informed consent obtained before beginning the survey. As the research was open to the public, it did not undergo formal ethics review; but all procedures adhered to the ethical principles outlined in the World Medical Association Declaration of Helsinki and complied with the UK Data Protection Act 1998 (the predecessor to GDPR, applicable to 2014 data collection), ensuring respect for individuals, integrity, and responsible data handling. The current study uses pre-existing data for an independent analysis unrelated to the original purpose. For this secondary analysis, anonymization was maintained by using an aggregated, non-identifiable dataset. Data security followed institutional standards, with the data stored exclusively on college-managed OneDrive for Business under restricted access.

A random sample of UK-resident respondents over 18 years of age received e-mail invitations with a survey link. Upon consenting, participants reported sociodemographic information and answered questions about social anxiety and other related concepts, which took approximately 20 min and for which they were compensated using incentives of their choice (cash, airline miles, or gift cards). After removing 17 participants who failed attention checks, the final sample comprised 222 participants aged 18–79 years (*M* = 50.55, *SD* = 16.65), 62.16% of whom self-identified as female. Regarding educational attainment, 9.01% of the participants had not completed O-levels, 28.38% had completed O-levels, 4.95% had completed post-16 vocational courses, 22.07% had completed A-levels, 26.13% had obtained undergraduate degrees, 8.56% had obtained postgraduate degrees, and 0.90% were still in education. The participants also reported annual household income, with 8.56% earning up to £9,999 per annum, 19.37% up to £19,999, 22.52% up to £29,999, 14.86% up to £39,999, 11.26% up to £49,999, 9.01% up to £74,999, 3.15% earning £75,000 or more, with 11.26% answering “don't know” or “rather not say”.

### 2.2 Measures

#### 2.2.1 Social anxiety

Social anxiety was measured with the Social Phobia Screening Questionnaire (Furmark et al., 1999). Participants rated their distress across 14 socially distressing situations, such as “speaking in front of a subgroup of people” and “maintaining a conversation with someone unfamiliar” on a 5-point scale (1 = *no distress*, 5 = *severe distress*). The diagnostic items were not administered, as the study focused on symptom levels in a community sample. Cronbach's alpha was 0.94.

#### 2.2.2 Depressive symptoms

Symptoms of depression were measured by the Depression Subscale from the 22-item Ruminative Responses Scale (Treynor et al., 2003). Prior research has interpreted this subscale as indicative of depressive symptoms, given its conceptual and item overlap with the widely used Beck Depression Inventory (Black and Pössel, 2013; Gustavson et al., 2020). Example items included “Think about how sad you feel” and “Think about how passive and unmotivated you feel”. Participants rated the frequency of their symptoms on a 4-point scale (1 = *almost never*, 4 = *almost always*). This subscale demonstrates strong convergent validity, correlating highly with the Beck Depression Inventory-II (*r* = 0.86; Berman et al., 2011) and with the Patient Health Questionnaire-9 (*r* = 0.69; Liu et al., 2023). Cronbach's alpha was 0.95.

#### 2.2.3 Daily affect

Daily affect was assessed by rating the extent to which the participants experienced positive (e.g., happy and relaxed) and negative affect (e.g., nervous and sad) at any point during the day on a 7-point scale (1 = *did not feel this way at all*, 7 = *felt this way very strongly*) (Nezlek and Kuppens, 2008). Cronbach's alphas were 0.90 for positive affect and 0.88 for negative affect.

#### 2.2.4 Reappraisal and suppression

Emotion regulation was measured with the Emotion Regulation Questionnaire, gauging reappraisal and suppression (Gross and John, 2003). Example items included “I control my emotions by changing the way I think about the situation I'm in” for reappraisal, and “I control my emotions by not expressing them” for suppression rated on a 7-point scale (1 = *not at all characteristic of me*, 7 = *very characteristic of me*). Cronbach's alphas were 0.90 for reappraisal and 0.86 for suppression.

#### 2.2.5 Rumination

Rumination was measured by the brooding and reflection subscales of the 22-item Ruminative Responses Scale (Treynor et al., 2003). Example items included “Go someplace alone to think about your feelings” and “Think about a recent situation, wishing it had gone better”. Participants rated the frequency of their ruminative responses to negative events or emotions on a 4-point scale (1 = *almost never*, 4 = *almost always*). Cronbach's alpha was 0.92.

### 2.3 Analytic strategy

Descriptive statistics, internal consistency, and bivariate correlations for all study variables were calculated using SPSS v29.0. Given the self-report nature of the data, we conducted a *post-hoc* Harman's single-factor test to assess the common method variance by entering all variables into a principal-components factor analysis with an unrotated factor solution (Podsakoff et al., 2003). Nine factors with eigenvalues greater than 1.0 emerged, accounting for 69.93% of the variance, with the first factor explaining 34.42%, suggesting that common method bias was not a serious concern in this study (Tehseen et al., 2017). To categorize participants into subgroups based on standardized mean scores for social anxiety and depressive symptoms, LPA were run using R v.4.2.2 with the *tidyLPA* package (Rosenberg et al., 2018). A series of analyses of variance (ANOVAs) compared the resulting profiles on daily affect and emotion regulation, following statistical approaches employed by recent LPA studies (Aonso-Diego et al., 2024; De Souza Marcovski and Miller, 2023; King et al., 2024). Q-Q plots were visually inspected to assess residual normality. Levene's tests were used to check for homogeneity of variances, and Welch's ANOVAs were applied if violated. To control for Type I errors, we employed the Bonferroni adjustment in conditions of equal variances and Tamhane's T2 for unequal variances when conducting pairwise multiple comparisons. We calculated omega squared (ω^2^) and Hedges' *g* as effect size estimates.

## 3 Results

### 3.1 Descriptives

The descriptives for key study variables are shown in Tables 1, 2. Skewness and kurtosis values indicated that all variables were approximately normally distributed. As expected, positive correlations between social anxiety and depressive symptoms emerged. Similar to previous literature, both social anxiety and depressive symptoms correlated negatively with daily positive affect and reappraisal, and positively with daily negative affect, suppression, and rumination.

**Table 1:** Descriptives for main study variables.

	**Mean**	***SD***	**Min**	**Max**	**Skewness**	**Kurtosis**
Social anxiety	2.41	0.88	1.00	5.00	0.45	−0.18
Depressive symptoms	1.93	0.68	1.00	4.00	0.71	−0.06
Daily positive affect	4.21	1.23	1.00	7.00	−0.34	0.05
Daily negative affect	2.49	1.26	1.00	6.17	0.77	−0.15
Reappraisal	4.15	1.20	1.00	7.00	−0.23	−0.03
Suppression	4.22	1.41	1.00	7.00	−0.30	−0.22
Rumination	1.87	0.66	1.00	4.00	0.80	0.28

**Table 2:** Correlations between main study variables.

	**1**	**2**	**3**	**4**	**5**	**6**	**7**
1 Social anxiety	1.00						
2 Depressive symptoms	0.53^***^	1.00					
3 Daily positive affect	−0.32^***^	−0.43^***^	1.00				
4 Daily negative affect	0.56^***^	0.63^***^	−0.38^***^	1.00			
5 Reappraisal	−0.20^**^	−0.15^*^	0.44^***^	−0.08	1.00		
6 Suppression	0.19^**^	0.18^**^	−0.04	0.23^***^	0.14^*^	1.00	
7 Rumination	0.52^***^	0.90^***^	−0.37^***^	0.62^***^	−0.07	0.16^*^	1.00

^***^p < 0.001, ^**^p < 0.01, ^*^p < 0.05.

### 3.2 Latent profiles of social anxiety and depressive symptoms

LPA assumes equal variances across latent profiles and local independence (i.e., uncorrelated indicators after accounting for latent profile membership) (Johnson, 2021) by default. Both assumptions, however, can be relaxed (Vermunt and Magidson, 2002). Drawing on Johnson (2021) and Pastor et al. (2007), we assessed four model configurations for each profile solution based on constraining or relaxing the variance and covariance assumptions. Specifically, in the default *profile-invariant diagonal* model configuration, variances for the same indicator are constrained to be equal across profiles, and indicators are not allowed to covary beyond their association within the same profile (i.e., all covariances are zero). In the *profile-varying diagonal* condition, variances can vary across profiles, while residual covariances are still fixed at zero. In the *profile-invariant unrestricted* condition, both variances and residual covariances are constrained to be equal across profiles. Finally, in the most flexible *profile-varying unrestricted* condition, variances and residual covariances are allowed to vary across profiles.

Based on Magson et al. (2022)'s findings of latent profiles of social anxiety and depressive symptoms, we tested up to five profile solutions across different model configurations. The selection of the final LPA solution considered both statistical fit indices and the substantive interpretability of the resulting profiles (Bauer, 2022). First, we used the Akaike information criterion (AIC) (Akaike, 1974), Bayesian information criterion (BIC) (Schwarz, 1978), and sample size-adjusted BIC (SABIC) (Sclove, 1987) based on the log likelihood (LL) of the models, with lower values indicating better fit. Second, we used the adjusted Lo–Mendell–Rubin likelihood ratio test (LMR-LRT) (Lo et al., 2001) and Bootstrap Likelihood Ratio Test (BLRT) (McLachlan and Peel, 2000) to assess the relative fit between *k*-profile and *k-1* profile models. Third, we estimated entropy, an indicator of classification accuracy, with values closer to 1 indicating clearer profile separations, and applied a cut-off of 0.7 (Reinecke, 2006; Van Zalk et al., 2020). Fourth, we checked the smallest profile size and excluded those with profiles representing less than 5% of the sample to avoid potentially unstable solutions (Nylund-Gibson and Choi, 2018). Moreover, we prioritized parsimonious profile solutions and visually inspected them to assess interpretability and distinguishability among profiles (Bauer, 2022). Fit indices and LPA results for all tested models across one- to five-profile solutions are presented in Table 3.

**Table 3:** Fit indices and LPA results for four model configurations across one- to five-profile solutions.

**Model**	**Profile**	**Npar**	**LL**	**AIC**	**BIC**	**SABIC**	**LMR**	**LMR_*p***	**BLRT**	**BLRT_*p***	**Entropy**	**% small**
Profile-invariant diagonal: *equal variances, zero covariances* (Model A)	1	4	−629.01	1,266.01	1,279.62	1,266.95	–	–	–	–	–	–
	2	7	−589.30	1,192.60	1,216.42	1,194.23	74.80	< 0.001	79.41	< 0.01	0.77	27.93
	3	10	−581.68	1,183.36	1,217.39	1,185.70	14.35	< 0.01	15.24	< 0.01	0.67	19.37
	**4**	**13**	–**574.21**	**1,174.43**	**1,218.67**	**1,177.47**	**14.06**	<**0.01**	**14.93**	**0.02**	**0.71**	**10.36**
	5	16	−574.19	1,180.38	1,234.83	1,184.12	0.04	1.00	0.05	0.56	0.58	0.00
Profile-varying diagonal: *varying variances, zero covariances* (Model B)	1	4	−629.01	1,266.01	1,279.62	1,266.95	–	–	–	–	–	–
	2	9	−573.68	1,165.36	1,195.99	1,167.46	104.22	< 0.001	110.65	< 0.01	0.77	30.63
	3	14	−555.53	1,139.05	1,186.69	1,142.32	34.20	< 0.001	36.31	< 0.01	0.66	24.32
	**4**	**19**	–**544.17**	**1,126.35**	**1,191.00**	**1,130.78**	**21.39**	<**0.001**	**22.71**	**0.03**	**0.71**	**6.31**
	5	24	−542.40	1,132.81	1,214.47	1,138.41	3.34	0.65	3.54	0.93	0.69	3.60
Profile-invariant unrestricted: *equal variances, equal covariances* (Model C)	1	5	−591.79	1,193.58	1,210.60	1194.75	–	–	–	–	–	–
	**2**	**8**	**−578.68**	**1,173.35**	**1,200.57**	**1,175.22**	**24.71**	<**0.001**	**26.23**	<**0.01**	**0.75**	**28.83**
	3	11	−580.60	1,183.19	1,220.62	1,185.76	−3.62	1.00	−3.84	0.98	0.68	2.70
	4	14	−571.90	1,171.79	1,219.43	1,175.06	16.39	< 0.001	17.40	0.02	0.54	0.00
	5	17	−573.65	1,181.31	1,239.15	1,185.28	−3.31	1.00	−3.52	0.94	0.55	0.00
Profile-varying unrestricted: *varying variances, varying covariances* (Model D)	1	5	−591.79	1,193.58	1,210.60	1,194.75	–	–	–	–	–	–
	2	11	−561.91	1,145.82	1,183.25	1,148.39	56.29	< 0.001	59.76	< 0.01	0.75	25.68
	3	17	−549.84	1,133.68	1,191.53	1,137.66	22.74	< 0.001	24.14	< 0.01	0.65	12.16
	**4**	**23**	–**537.46**	**1,120.92**	**1,199.18**	**1,126.29**	**23.33**	<**0.001**	**24.77**	<**0.01**	**0.70**	**8.11**
	5	29	−531.71	1,121.42	1,220.09	1,128.19	10.83	0.09	11.50	0.40	0.67	6.31
Comparison	A-4	13	−574.21	1,174.43	1,218.67	1,177.47	14.06	< 0.01	14.93	0.02	0.71	10.36
	B-4	19	−544.17	1,126.35	1,191.00	1,130.78	21.39	< 0.001	22.71	0.03	0.71	6.31
	C-2	8	−578.68	1,173.35	1,200.57	1,175.22	24.71	< 0.001	26.23	< 0.01	0.75	28.83
	D-4	23	−537.46	1,120.92	1,199.18	1,126.29	23.33	< 0.001	24.77	< 0.01	0.70	8.11

Npar, number of free parameters; LL, log likelihood; AIC, Akaike information criterion; BIC, Bayesian information criterion; SABIC, sample size-adjusted BIC; LMR, Lo-Mendell-Rubin likelihood ratio test; LMR_p, p-value of the LMR; BLRT, bootstrapped likelihood ratio test; BLRT_p, p-value of the BLRT; % small, percentage of participants in the smallest profile; boldened row indicates the indicate the selected solution in each model configuration based on multiple fit indices.

In both *profile-invariant diagonal* (Model A) and *profile-varying unrestricted* conditions (Model D), the AIC and SABIC reached their lowest values for the 4-profile solution, while the BIC was lowest for the 2-profile solution. The LMR-LRT and BLRT were non-significant for the 5-profile solution, in preference of the 4-profile model. The entropy was highest for the 2-profile solution and adequate for the 4-profile solution. Accordingly, we chose the 4-profile solution (i.e., A-4 and D-4) for both model configurations. In the *profile-varying diagonal* condition (Model B), similarly, the AIC and SABIC were lowest for the 4-profile solution, and the BIC was lowest for the 3-profile solution. The LMR-LRT and BLRT became non-significant for the 5-profile solution. The entropy was optimal for the 2-profile solution and adequate for the 4-profile solution. We thus chose the 4-profile solution (i.e., B-4) for this model configuration. In the *profile-invariant unrestricted* condition (Model C), the AIC and SABIC were lowest for the 4-profile solution, and the BIC was lowest for the 2-profile solution. The LMR-LRT and BLRT were significant for the 2- and 4-profile solutions. However, only the 2-profile solution achieved an adequate level of entropy, leading us to select this solution (i.e., C-2).

Furthermore, we compared the four selected solutions from different model configurations. Comparisons of the AIC, BIC, and SABIC revealed that the B-4 and D-4 outperformed the A-4 and C-2. All four profiles displayed comparable entropy, indicating moderate classification certainty, with the C-2 slightly better than the other solutions. In addition, we inspected the solution interpretability, with the B-4 and D-4 demonstrating similar participant patterns. In both solutions, the profile characterized by low social anxiety and depressive symptoms accounted for less than 10% of the participants, which substantially deviates from the approximately 60-80% representing low psychological distress in adult community samples (Adzrago et al., 2025; Gondek et al., 2022; Lee et al., 2015). In the C-2, the classification into two profiles was primarily driven by the level of depressive symptoms. Therefore, the representativeness and substantive value of the three solutions (i.e., B-4, D-4, and C-2) were inadequate. In contrast, the A-4 provided substantively interpretable profiles and reproduced four latent profiles identified in Magson et al. (2022). Also, it yielded relatively more balanced profile sizes. Therefore, the 4-profile solution of the profile-invariant diagonal model configuration (i.e., A-4) was found to be preferable and was used in the subsequent analyses.

**Figure 1:**
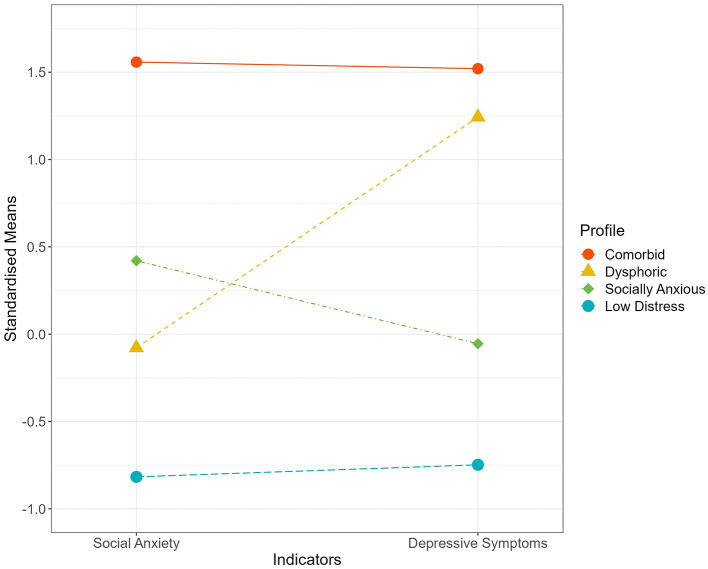
Standardized means of social anxiety and depressive symptoms for the four profiles. Points and connecting lines illustrate how each profile differs in symptom severity across the two indicators, with higher values indicating greater severity. The Comorbid profile (12.61%) exhibited the highest levels of both social anxiety and depressive symptoms. In contrast, the Dysphoric profile (10.36%) was marked by moderate depressive symptoms alongside relatively low social anxiety. The Socially Anxious profile (36.94%) displayed moderate social anxiety but minimal depressive symptoms. Lastly, the Low Distress profile (40.09%) reflected the lowest levels of both symptoms.

The standardized means of social anxiety and depressive symptoms across the profiles are displayed in Figure 1. The largest subgroup comprised individuals with the lowest symptom levels of social anxiety and depression (40.09% of the sample; *n* = 89). Consistent with Magson et al. (2022), we labeled this subgroup as “Low Distress”, reflecting the minimal symptom burden across both domains. The second largest subgroup, comprising 36.94% of the sample (*n* = 82), showed moderate social anxiety but lower depressive symptoms. This subgroup was labeled as “Socially Anxious” to reflect the relative prominence of social anxiety in their symptom configuration. We avoided using “socially phobic”, as social phobia is the older term for “social anxiety disorder” (Bruce et al., 2012) and thus implies a formal disorder with pathological severity inconsistent with this subgroup's predominantly subclinical symptom levels. We also avoided “shyness”, given its strong temperament origins, lack of clinical specificity, and ongoing debate about its distinction from social anxiety (Brook and Willoughby, 2019). Additionally, 10.36% of the sample (*n* = 23) showed moderate depressive symptoms but lower social anxiety. This subgroup was labeled as “Dysphoric” to reflect the relative prominence of depressive symptoms. We used “dysphoric” rather than “depressive”, as dysphoria is a common term to denote depression at the mild or subclinical level (Catrambone et al., 2021; Ridout et al., 2025) consistent with this subgroup's symptom presentation. Finally, 12.61% of the sample (*n* = 28) showed the highest levels of social anxiety and depressive symptoms. This subgroup was labeled as “Comorbid” to reflect the co-occurrence of heightened symptoms in both domains. It should be noted that the descriptions of low, moderate, and high symptom levels here were relative to the distribution of scores in the current sample and do not reflect clinical diagnostic classifications. Clinical cut-offs were not referred to, as our primary aim was to uncover latent profiles of social anxiety and depressive symptoms in a community sample.

### 3.3 Between-profile differences in daily affect and emotion regulation

Chi-square tests of independence revealed no significant differences in gender [$\chi_{\left(3\right)}^{2}$ = 3.64, *p* = 0.30], educational attainment [$\chi_{\left(15\right)}^{2}$ = 23.38, *p* = 0.08], or household income [$\chi_{\left(18\right)}^{2}$ = 17.18, *p* = 0.51] between the four profiles, though significant age differences (*F*_(3, 218)_ = 7.18, *p* < 0.001, ω^2^ = 0.08 [0.02, 0.16]) emerged with an ANOVA. We conducted ANCOVAs with age as a covariate to test for emotional functioning differences between profiles. The significant results from the ANOVAs remained significant after controlling for age. For simplicity, we reported the ANOVA results.

**Figure 2:**
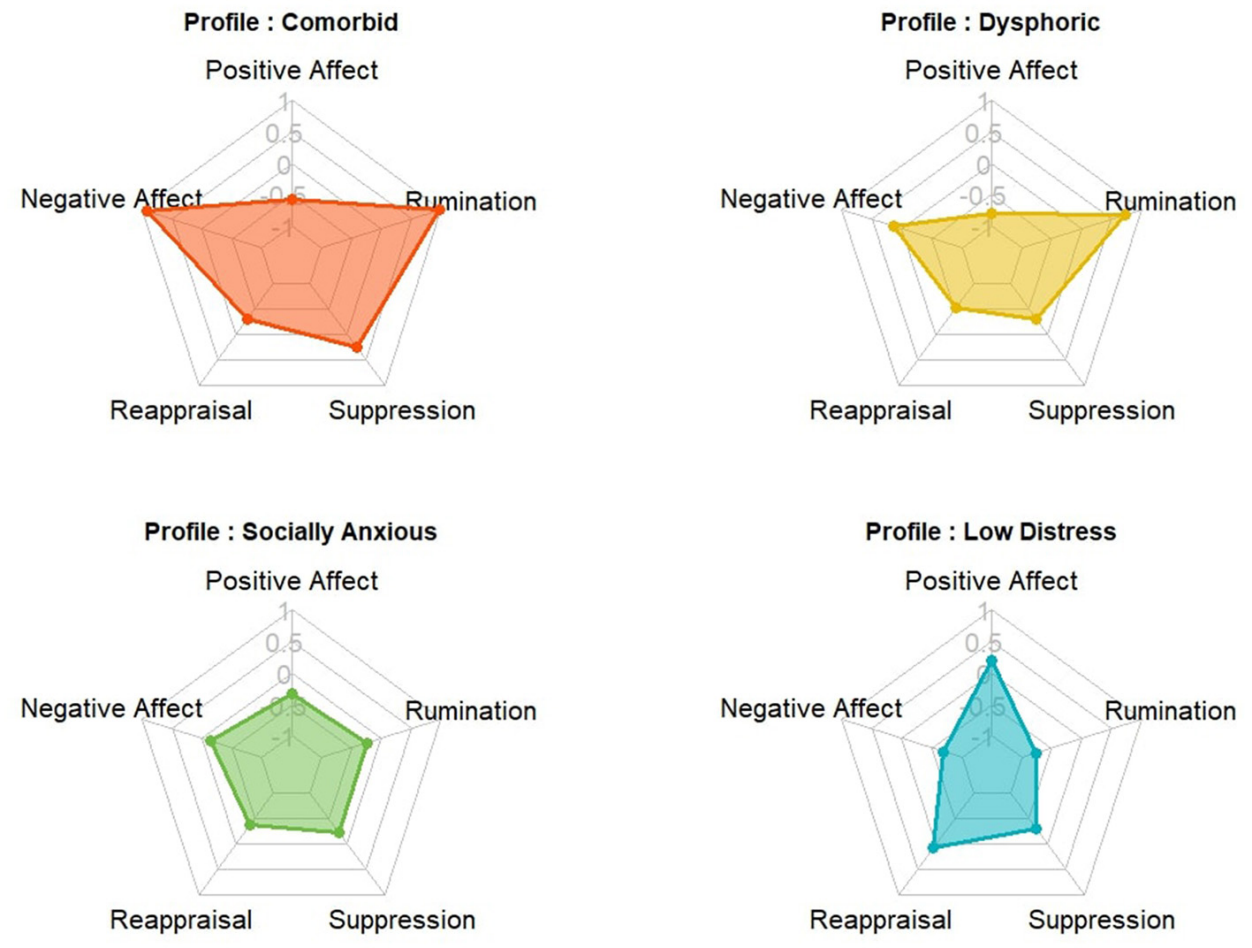
Radar charts of standardized means for emotional functioning across the four profiles. Each chart illustrates the distinct pattern of the five indicators of emotional functioning characterizing each profile. The Comorbid profile showed the greatest emotional dysfunction, with more negative affect, less positive affect, and higher emotion dysregulation. The Low Distress profile functioned best emotionally. The Socially Anxious and Dysphoric profiles fell in between, while the Dysphoric profile reported lower positive affect and more rumination.

Profile mean-level comparisons of daily affect and emotion regulation are shown in Table 4 and Figure 2. There were significant differences among the profiles in daily positive (*F*_(3, 218)_ = 17.13, *p* < 0.001, ω^2^ = 0.18 [0.10, 0.27]) and negative affect ($Welch^{\prime }sF_{\left(3,63.22\right)}$ = 43.35, *p* < 0.001, ω^2^ = 0.36 [0.24, 0.47]). Pairwise comparisons showed that the Low Distress subgroup had significantly higher daily positive affect than all other subgroups. The Comorbid subgroup had similar positive affect levels to the Dysphoric and Socially Anxious subgroups. Although the Dysphoric subgroup reported lower positive affect than the Socially Anxious subgroup, this difference was not significant.

**Table 4:** Differences in emotional functioning among the identified profiles.

	**Comorbid (*n* = 28)**	**Dysphoric (*n* = 23)**	**Socially anxious (*n* = 82)**	**Low distress (*n* = 89)**	**Pairwise comparisons**	**Hedges' *g* [95% CI]**
Daily positive affect	3.63 (1.31)	3.30 (1.30)	4.00 (1.04)	4.81 (1.06)	Comorbid = Dysphoric	0.25 [−0.31, 0.81]
					Comorbid = Socially Anxious	−0.33 [−0.76, 0.10]
					Comorbid < Low Distress^***^	−1.04 [−1.49, −0.60]
					Dysphoric < Socially Anxious^†^	−0.63 [−1.10, −0.16]
					Dysphoric < Low Distress^***^	−1.34 [−1.84, −0.85]
					Socially Anxious < Low Distress^***^	−0.76 [−1.07, −0.45]
Daily negative affect	4.26 (1.36)	3.03 (0.94)	2.58 (1.01)	1.71 (0.74)	Comorbid > Dysphoric^**^	1.02 [0.43, 1.61]
					Comorbid > Socially Anxious^***^	1.51 [1.03, 1.98]
					Comorbid > Low Distress^***^	2.74 [2.19, 3.30]
					Dysphoric = Socially Anxious	0.45 [−0.02, 0.92]
					Dysphoric > Low Distress^***^	1.67 [1.16, 2.17]
					Socially Anxious > Low Distress^***^	0.98 [0.67, 1.30]
Reappraisal	4.01 (1.50)	3.65 (1.18)	3.87 (1.00)	4.57 (1.15)	Comorbid = Dysphoric	0.26 [−0.30, 0.81]
					Comorbid = Socially Anxious	0.12 [−0.31, 0.55]
					Comorbid = Low Distress	−0.45 [−0.88, −0.02]
					Dysphoric = Socially Anxious	−0.21 [−0.67, 0.26]
					Dysphoric < Low Distress^**^	−0.79 [−1.26, −0.32]
					Socially Anxious < Low Distress^***^	−0.65 [−0.95, −0.34]
Suppression	5.01 (1.39)	4.04 (1.77)	4.19 (1.10)	4.04 (1.51)	Comorbid = Dysphoric	0.60 [0.04, 1.17]
					Comorbid > Socially Anxious^*^	0.69 [0.25, 1.13]
					Comorbid > Low Distress^*^	0.65 [0.22, 1.09]
					Dysphoric = Socially Anxious	−0.11 [−0.58, 0.35]
					Dysphoric = Low Distress	0.004 [−0.46, 0.46]
					Socially Anxious = Low Distress	0.11 [−0.19, 0.42]
Rumination	2.84 (0.51)	2.64 (0.47)	1.83 (0.42)	1.41 (0.36)	Comorbid = Dysphoric	0.41 [−0.16, 0.97]
					Comorbid > Socially Anxious^***^	2.27 [1.74, 2.79]
					Comorbid > Low Distress^***^	3.53 [2.90, 4.15]
					Dysphoric > Socially Anxious^***^	1.86 [1.33, 2.39]
					Dysphoric > Low Distress^***^	3.14 [2.53, 3.76]
					Socially Anxious > Low Distress^***^	1.08 [0.75, 1.40]
Age	41.54 (16.41)	42.39 (16.26)	51.22 (16.08)	54.88 (15.70)	Comorbid = Dysphoric	−0.05 [−0.61, 0.51]
					Comorbid < Socially Anxious^*^	−0.59 [−1.03, −0.16]
					Comorbid < Low Distress^***^	−0.83 [−1.27, −0.40]
					Dysphoric = Socially Anxious	−0.54 [−1.01, −0.07]
					Dysphoric < Low Distress^**^	−0.78 [−1.26, −0.31]
					Socially Anxious = Low Distress	−0.23 [−0.53, 0.07]

Values for each profile represent Means (SDs). For pairwise comparisons, the Bonferroni adjustment was used when variances were equal, while Tamhane's T2 was used for unequal variances. Hedges' g were calculated as effect size estimates.

^***^p < 0.001, ^**^p < 0.01, ^*^p < 0.05,^†^p < 0.1.

For negative affect, the Comorbid subgroup reported the highest levels, exceeding both the Dysphoric and Socially Anxious subgroups, which had similar levels. The Low Distress subgroup had the lowest daily negative affect.

There were also significant differences among the profiles in emotion regulation, including reappraisal (*F*_(3, 218)_ = 7.16, *p* < 0.001, ω^2^ = 0.08 [0.02, 0.16]), suppression ($Welch^{\prime }sF_{\left(3,64.25\right)}$ = 3.48, *p* = 0.02, ω^2^ = 0.03 [0.00, 0.10]), and rumination (*F*_(3, 218)_ = 113.66, *p* < 0.001, ω^2^ = 0.60 [0.53, 0.67]). The Low Distress subgroup used reappraisal significantly more than the Dysphoric and Socially Anxious subgroups, which had similar levels. The Comorbid subgroup fell in between, with no significant differences from the others.

For suppression, the Comorbid subgroup reported significantly higher levels than the Socially Anxious and Low Distress subgroups. However, there were no significant differences in suppression among the Dysphoric, Socially Anxious, and Low Distress subgroups. Although the Comorbid subgroup had a noticeably higher mean, it did not differ significantly from the Dysphoric subgroup, likely due to smaller sample sizes for these two subgroups.

Both the Comorbid and Dysphoric subgroups reported high levels of rumination, significantly more than the Socially Anxious and Low Distress subgroups. The Socially Anxious subgroup also ruminated significantly more than Low Distress subgroup.

## 4 Discussion

This study used LPA to investigate the co-occurrence of social anxiety and depressive symptoms in a UK adult sample. In line with our hypotheses and replicating Magson et al. (2022)'s findings on adolescents, we identified four profiles with varying symptom levels: those with high social anxiety and depressive symptoms (Comorbid), those with moderate depressive symptoms and low social anxiety (Dysphoric), those with moderate social anxiety and low depressive symptoms (Socially Anxious), and those with low levels of both (Low Distress). However, the profile distributions in our study differed from those observed in Magson et al. (2022). The Comorbid subgroup (12.61%) was slightly larger than the Dysphoric subgroup (10.36%), whereas it was the smallest profile in the adolescent sample with a prevalence of 8%. The largest, Low Distress subgroup (40.09%) was less prevalent than the equivalent profile in adolescents, which constituted more than half of that sample. Although under 25% among adolescents, the Socially Anxious subgroup accounted for 36.94% of our participants, nearly comparable to the Low Distress subgroup.

The identified four profiles align with clinical observations associating comorbidity with greater symptom severity compared to when social anxiety and depressive disorders occur alone (Adams et al., 2018; Koyuncu et al., 2014). These profiles differed in the main types of symptoms they experienced, not just in severity. This is consistent with the moderate correlation (*r* = 0.53) between social anxiety and depressive symptoms in our study, and diverges from research identifying profiles of generalized anxiety and depressive symptoms characterized by unidimensional severity levels (Dai et al., 2024; Wang et al., 2021). Our identification of non-parallel profiles supports viewing social anxiety and depression as distinct constructs with shared features, adding to the debate on whether anxiety and depression are a unitary construct or distinct entities (Endler et al., 2003; McElroy et al., 2018; Prenoveau et al., 2011). Regarding the differences in profile prevalence compared to the adolescent study, they may be related to factors such as age, culture, and other sociodemographic characteristics; but the differences may also stem from the modest sample size and/or possible self-selection bias in recruitment, oversampling participants who were relatively more socially anxious.

Furthermore, we found significant between-profile differences in emotional functioning, measured by daily affect and emotion regulation. Notably, all significant differences in emotional functioning between profiles reached above medium effect sizes (Hedges' *g* > 0.5, see Table 4), demonstrating the distinctiveness and meaningfulness of the four identified profiles. Overall, the Comorbid subgroup showed more pronounced emotional dysfunction than the other three subgroups, whereas the Low Distress subgroup showed the best emotional functioning.

Specifically, as expected, the Low Distress subgroup reported the highest positive affect. The Comorbid and Dysphoric subgroups had similar levels of positive affect, whereas the Socially Anxious subgroup had marginally higher positive affect than the Dysphoric subgroup. These results indicate that while all profiles were affected by some form of distress—whether depression or social anxiety—depressive symptoms had a stronger or more consistent negative impact on positive affect. This aligns with extant literature showing that depressive symptoms are more strongly associated with reduced positive affect than social anxiety (Chin et al., 2023; Watson and Naragon-Gainey, 2010). Additionally, the Comorbid subgroup had higher negative affect compared to the Dysphoric and Socially Anxious subgroups, which reported similar levels. The Low Distress subgroup had the lowest negative affect. As has been well documented (Langer and Rodebaugh, 2014; Watson and Naragon-Gainey, 2010), our results reflect that both social anxiety and depressive symptoms are strongly associated with negative affect, with the symptom co-occurrence further intensifying negative emotional experiences.

The Low Distress subgroup reported more cognitive reappraisal, consistent with a large literature recognizing reappraisal as an adaptive regulation strategy (Stover et al., 2024). Interestingly, the Comorbid subgroup used reappraisal relatively frequently, with no statistical difference compared to the Low Distress subgroup, suggesting an ineffective use of reappraisal in this subgroup. Cognitive reappraisal relies on executive resources, with its efficacy dropping under high cognitive load (Gan et al., 2017). We speculate that the dual burden of depression-related repetitive negative thinking (Yang et al., 2017) and social anxiety-related attention bias (Neophytou and Panayiotou, 2024) may impose a sustained high cognitive load on the Comorbid subgroup, thereby limiting the executive resources available for effective reappraisal. There were also no differences between the Comorbid, Dysphoric, and Socially Anxious subgroups, aligning with Aldao and Nolen-Hoeksema (2012)'s finding no direct association between the use of reappraisal and psychopathology.

The Comorbid subgroup had the highest levels of emotional suppression. Despite reaching a medium effect size (*g* = 0.60), the difference in suppression between the Comorbid and Dysphoric subgroups was not statistically significant, which was plausibly due to the small subgroup sizes resulting in insufficient power to detect the difference. Both the Socially Anxious and Dysphoric subgroups reported using suppression at levels comparable to the Low Distress subgroup. These results are partially consistent with Dryman and Heimberg (2018)'s review noting a mixed relationship between depression and suppression while linking social anxiety to an overreliance on suppression. We speculate that distinct profile-specific regulatory patterns for suppression underlie these findings. Suppression in the Socially Anxious subgroup may be strategic and specific to socially evaluative contexts (Spokas et al., 2009), while the Dysphoric subgroup's attenuated emotional reactivity and insensitivity to emotional contexts may naturally reduce the need for suppression (Ellis et al., 2009). However, the elevated suppression for the Comorbid subgroup suggests that symptom interactions may create unique regulatory demands; Gökdag et al. (2024) found that social anxiety increased emotional suppression, which in turn exacerbated depressive symptoms, establishing a pathological cascade.

In addition, rumination levels corresponded to symptom severity across the profiles. The Comorbid subgroup reported the highest levels of rumination, followed by the Dysphoric, Socially Anxious, and Low Distress subgroups in descending order. While numerically higher in the Comorbid subgroup, no statistical difference in rumination was found compared to the Dysphoric subgroup, plausibly due to small subgroup sizes. The overall trend aligns with Arditte Hall et al. (2019) showing that participants diagnosed with social anxiety or major depressive disorder used more rumination than healthy controls, with the highest levels reported in those with comorbid conditions. The severity-dependent trend supports treating rumination as a crucial transdiagnostic factor in depression and social anxiety (Hsu et al., 2015; McLaughlin and Nolen-Hoeksema, 2011), highlighting the promise of transdiagnostic interventions for emotional disorders directly targeting rumination (Tulbure et al., 2025).

This study has several limitations. First, the sample size was relatively small. Given that the estimated LPA models included only two indicators and were expected to derive a relatively small number of profiles, the sample size was deemed adequate to detect the correct number of profiles (Nylund-Gibson and Choi, 2018). However, the small sample size limited the robustness of our profile prevalence estimates. It also posed challenges for cross-profile comparisons, especially as the profile prevalences were unequal, with small subgroup sizes for the comorbid and dysphoric profiles. Using G^*^Power 3.1.9.7 (Faul et al., 2009), a sensitivity power analysis for the smallest subgroup pair (*n*_1_ = 22, *n*_2_ = 23; α = 0.05; power = 80%) indicated a minimum detectable effect size of Cohen's *d* = 0.85 (equivalent to Hedges' *g* = 0.84), indicating that only large effects could be reliably identified. It is thus necessary to replicate our results and confirm the profile prevalences in larger samples. Second, depressive symptoms were assessed by the depression subscale of the Ruminative Responses Scale. We acknowledge that this subscale may not capture the full spectrum of depressive symptoms, and future research should consider using dedicated measures, such as the Beck Depression Inventory-II (Beck et al., 1996), the Patient Health Questionnaire-9 (Kroenke et al., 2001), and the Center for Epidemiologic Studies-Depression (Radloff, 1977). Third, self-report measures may introduce common method bias. While Harman's single-factor test indicated this bias was not a serious concern in our data, future research can address this limitation directly by incorporating behavioral or physiological measures and employing a multi-trait multi-method design.

Fourth, the self-selected, public-facing nature of recruitment may have skewed prevalence estimates by oversampling participants with relatively higher social anxiety symptoms. Therefore, while the four-profile solution is likely robust, the observed profile prevalences may not fully generalize to the broader UK population from which the study sample was drawn. Future research should consider using probability-based recruitment strategies. Additionally, symptom expression within the UK cohort may differ from other cultural contexts, as the experience and reporting of psychological distress are culturally shaped (Kohrt et al., 2014). Cross-cultural replications, using culture-sensitive symptom measures to ensure conceptual equivalence, are needed to validate the current four-profile solution across diverse settings. Furthermore, these profiles were identified based on self-reported symptoms rather than diagnoses. Future research should bridge the gap by validating profiles in clinical samples and testing predictive power for later disorder onset. Fifth, the reliance on cross-sectional data precluded the investigation of stability and potential transitions across profiles over time. Magson et al. (2022) on adolescents found fairly stable profiles of social anxiety and depressive symptoms over time using Latent Transition Analysis; future work should employ similar longitudinal analyses to determine whether these profiles maintain stability into adulthood. The age differences among profiles, with individuals in the low distress profile being the oldest, also suggest the need for longitudinal studies to distinguish cohort effects from developmental changes associated with aging. Moreover, longitudinal studies could strengthen our findings by clarifying the timing of emotional functioning and psychiatric symptoms. Finally, our data were collected in 2014, prior to the COVID-19 pandemic. While research shows an increased prevalence of depressive and anxiety disorders during the pandemic (Santomauro et al., 2021), there is also evidence indicating that the lockdown had only modest overall effects on mental health, and that multinational suicide rates remained largely stable before and during the pandemic (Prati and Mancini, 2021). This aligns with research on psychological resilience, which consistently demonstrates that disasters transiently elevate population-level distress rather than causing permanent shifts (Bonanno et al., 2024). Our study offers a valuable pre-pandemic benchmark that future research can use to assess the stability and generalizability of our findings in post-pandemic contexts.

The study also has several methodological strengths. It is the first of its kind to examine the latent heterogeneity of co-occurring social anxiety and depressive symptoms in an adult community sample. We employed LPA, a person-oriented approach that fundamentally diverges from traditional variable-oriented methods which assume population homogeneity (Nylund, 2007). This person-oriented approach enabled us to identify distinct subgroups, each with unique symptom configurations that would be obscured or averaged into misleading patterns by variable-oriented analyses. Moreover, LPA's model-based framework provides statistical rigor unavailable in traditional clustering. It incorporates measurement error through its probability-based model estimation and uses objective fit indices and entropy metrics to determine the optimal profile solution without arbitrary cutoffs (Pastor et al., 2007), thus ensuring replicable subgroup identification. Also, LPA is well-suited to detecting heterogeneity in community samples (Williams and Kibowski, 2015) which span a broad continuum of symptom severity and can uncover subgroups that reflect real-world distributions (e.g., profiles with subclinical symptoms). Building on profile identification, we further investigated profile-specific pathways linking symptom configurations to transdiagnostic emotional processes. By identifying practically meaningful differences in emotional functioning across profiles, we validated the theoretical and functional significance of LPA-derived symptom profiles.

This study helps explain the variation in how symptoms of social anxiety and depression occur together. The four identified profiles show distinct and non-parallel symptom patterns, avoiding the “salsa effect”—a pitfall in LPA that involves statistically categorizing data into unidimensional levels (e.g., low, medium, and high severity) rather than meaningful subgroups (Sinha et al., 2021). Moreover, replicating this four-profile solution in the current UK adult sample and a previously studied Swedish adolescent sample (Magson et al., 2022) demonstrates the validity of these profiles and their consistency across populations and cohorts. Our results provide further insights into how individuals within each symptom profile experience and regulate their daily emotions. This aligns with the dimensional, transdiagnostic view of “emotional disorder” (Bullis et al., 2019), defined by: (1) frequent and intense negative emotions, (2) negative appraisals and aversive reactions to these emotions, and (3) significant efforts to suppress or avoid them. Our results also support the use of transdiagnostic interventions to address a range of anxiety, depression, and related distress by targeting common underlying factors, such as emotional functioning, instead of relying on separate treatment protocols for specific diagnoses (Bullis et al., 2019; Cuijpers et al., 2023). The identified profiles illustrate how such transdiagnostic approaches may be tailored. For example, the Comorbid profile suggests that future intervention research on co-occurring symptoms may target reducing rumination and suppression while enhancing reappraisal efficacy. The Dysphoric profile points to researching interventions that address low positive affect alongside rumination in subclinical depression. Finally, although replication in a more representative sample is needed, our findings highlight a significant proportion of participants within the Socially Anxious profile. Compared to the Low Distress profile, the Socially Anxious profile demonstrated medium-to-large deficits (Hedges' *g* > 0.5) across affect and regulation domains, highlighting the need for further research and clinical attention to this subgroup. In sum, our findings underscore the potential of tailored transdiagnostic interventions to more effectively address the diverse emotional and regulatory needs of individuals across the internalizing spectrum.

## Data Availability

The data analyzed in this study is subject to the following licenses/restrictions: the raw data analyzed in this study are not publicly available due to absence of participant consent for data sharing at the time of data collection. Requests to access these datasets should be directed to JZ (for descriptive statistics and other summary data upon request for research purposes such as meta-analysis).
